# Contribution of phylogenetics to understanding the evolution and epidemiology of dengue virus

**DOI:** 10.1002/ame2.12283

**Published:** 2022-10-17

**Authors:** Xi Yu, Gong Cheng

**Affiliations:** ^1^ Tsinghua‐Peking Center for Life Sciences, School of Medicine Tsinghua University Beijing China; ^2^ Institute of Infectious Diseases Shenzhen Bay Laboratory Shenzhen China; ^3^ Institute of Pathogenic Organisms Shenzhen Center for Disease Control and Prevention Shenzhen China; ^4^ School of Life Sciences Tsinghua University Beijing China

**Keywords:** dengue virus (DENV), evolution, flavivirus, phylogenetics

## Abstract

Dengue virus (DENV) is one of the most important arboviral pathogens in the tropics and subtropics, and nearly one‐third of the world's population is at risk of infection. The transmission of DENV involves a sylvatic cycle between nonhuman primates (NHP) and *Aedes* genus mosquitoes, and an endemic cycle between human hosts and predominantly *Aedes aegypti*. DENV belongs to the genus *Flavivirus* of the family *Flaviviridae* and consists of four antigenically distinct serotypes (DENV‐1‐4). Phylogenetic analyses of DENV have revealed its origin, epidemiology, and the drivers that determine its molecular evolution in nature. This review discusses how phylogenetic research has improved our understanding of DENV evolution and how it affects viral ecology and improved our ability to analyze and predict future DENV emergence.

## Graphical abstract

The ecology of DENV is a sylvatic cycle that alternates between non‐human primates and the *Aedes* genus mosquitoes. This sylvatic life cycle may extend to human hosts and subsequently establish an endemic cycle dominated primarily by *Aedes aegypti*. The drivers of DENV evolution and diversity include replication errors, purifying selection pressure, positive selection pressure, lineage shift and replacement, and recombination.

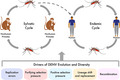

## 1 INTRODUCTION

Dengue virus (DENV) is the cause of high morbidity and mortality annually, and the global burden of DENV is increasing. Dengue virus is currently prevalent in 128 countries, and has now spread to non‐tropical areas such as Europe.^1^, ^2^ Dengue virus is also resurfacing in countries where native DENV has been non‐existent for decades.^3^, ^4^ Dengue epidemics can place a significant burden on limited health care systems, with an estimated 390 million infections, 500 000 hospitalizations, and 20 000 deaths per year.^2^, ^5^, ^6^ Globalization, transportation, trade, climate change, and the expanding range of the *Aedes* mosquito vector are expected to further increase the outbreak and spread of the virus to new geographic areas.^7^ Despite advances in the research and development of DENV vaccines, clinical management and vector control continue to form the basis of DENV prevention and control. While clinical treatment has effectively reduced the mortality caused by DENV in many cases, vector prevention and control efforts have been relatively ineffective.^6^

The persistent DENV burden has largely resulted from the circulation of four different DENV serotypes with overlapping endemic areas and partial host cross‐protection.^8^, ^9^ Thus, understanding DENV evolution through advances in genome sequencing, phylogenetics, bioinformatics, and other evolutionary and computational biology approaches offers a valuable opportunity to improve surveillance and response to DENV outbreaks. Here, we discuss the evolution and epidemiology of DENV and illustrate how evolutionary analyses can be utilized as effective tools to control this pathogen.

## 2 DENGUE VIRUS

DENV is an enveloped virus with a genome consisting of a single‐stranded RNA approximately 10 to 11 kbp in length. The dengue genome consists of three structural and seven non‐structural coding genes, all translated into a single polypeptide with two untranslated regions (UTR) at the 3′ ends and the 5′ ends. Examination of the structures of the viral proteins has provided insights into their effects on protein function, including the dynamics of viral morphology, the availability of binding sites, their roles in viral replication and pathogenesis, and variations in identified epitopes. Widely used approaches to study viral diversity rely on the analysis of individual amino acid positions of an alignment. A robust method based on Shannon's entropy is used to measure the degree of conservation and variability of peptides of desired length and infer their evolutionary stability. For immunological applications, the entropy measure for viral sequences is based on overlapping nonamer peptides. The entropy of the DENV proteome provides useful insight into viral evolution and diversity.^10^, ^11^

DENV belongs to the *Flavivirus* genus, which also includes several other medically important arboviruses such as Zika virus (ZIKV), yellow fever virus (YFV), Japanese encephalitis virus (JEV) and West Nile virus (WNV).^12^ Global transmission is achieved via humans as both reservoirs and amplifying hosts.^13^ DENV is maintained in its natural reservoir, non‐human primates and the *Aedes* genus mosquitoes, through the sylvatic cycle.^14^ This sylvatic life cycle may extend to human hosts and subsequently establish an endemic cycle dominated primarily by *Aedes aegypti*.^9^, ^15^, ^16^ Compared to other arboviruses in the *Flaviviridae* family, such as WNV and JEV, DENV has a limited host range in vertebrates.^16^, ^17^

DENV is classified into four serotypes, DENV‐1‐4. Within each DENV serotype, clusters of DENV viruses with nucleotide sequence divergence not >6% within a given genome region are further defined as genotypes.^9^, ^18^ Each of the four DENV serotypes is thought to originate from its own sylvatic cycle.^17^, ^19^ Sylvatic DENV strains have been isolated mainly from non‐human primates (NHP). However, the spill‐over to the endemic cycle is thought to continue occurring.^9^, ^12^ There is certainly considerable diversity among unsampled sylvatic DENV. It was reported that a sporadic sylvatic DENV strain causing infection in humans in Borneo in 2007 was identified as a ‘fifth dengue serotype’ because the viral sequence differed from all known DENV strains.^20^

Dengue fever is characterized by symptoms ranging from asymptomatic infections to a self‐limiting dengue fever and then to more severe systemic symptoms including capillary leakage, hemorrhage, shock, and death.^21^ It is worth noting that the percentage of dengue infections that develop to the onset of such severe symptoms is generally low.^21^ In general, infection by one dengue serotype leads to lifelong protection against that same serotype, short‐term protection against all serotypes for 1–3 years, and subsequent protection against more severe disease upon contact with other serotypes due to a process called antibody‐dependent enhancement (ADE).^22^, ^23^ ADE of viral infection is a phenomenon in which virus‐specific antibodies promote viral entry and, in some cases viral replication, in monocytes/macrophages and granulocytic cells through interaction with Fc and/or complement receptors. It is hypothesized that this is an evolutionary mechanism that DENV developed to avoid simultaneous competition of four serotypes against the same host population.^9^ The risk of developing severe disease increases with the second infection and decreases after the second infection.^24^

## 3 CHARACTERIZATION OF THE EVOLUTION OF DENV

DENV genome sequencing of human serum or vector samples is most commonly used in the molecular epidemiological analysis of DENV.^25^ The relatively small size of the DENV genome has the advantage of allowing next‐generation sequencing (NGS) approaches, and public DENV genome libraries (e.g., GenBank) have grown rapidly over the past years. The quality of these sequence data varies and requires multistep quality control before finally being used for analysis.^26^

Subsequently, evolutionary analysis of consensus DENV genomes has typically been relegated to phylogenetics and phylogenomics,^27^, ^28^ where phylogenetic trees are extrapolated to explain evolution as a function of interviral relationships and genetic distance.^28^ As DENV evolution occurs on a scale which often matches that of DENV transmission and epidemics, genetic variances among common DENV strains can often be detected within a few weeks, especially when genome‐wide data are used.^29^, ^30^, ^31^

In recent years, Bayesian phylogenetic methods have been widely applied using software such as BEAST (Bayesian Evolutionary Analysis of Sampling Trees).^32^ Bayesian methods also allow for the construction of complex evolutionary models, such as those describing changes in the rate of molecular evolution, population dynamics, and spatial migration. Information on geographic origin can also be inferred if available for the sample.^26^

## 4 SYLVATIC AND ENDEMIC DENV

Comparative analysis of the evolution of sylvatic and endemic DENV sequences has provided important insights into DENV biology. In DENV‐1, −2, and − 4, the evolution and epidemiology of sylvatic and endemic virus lineages remain distinct. Phylogenetic evidence has revealed that endemic DENV‐1, −2, and − 4 strains did not arise from a single ancestor but from independent sylvatic cycles. No data are available on the sequence of sylvatic DENV‐3, but this serotype is also thought to have evolved from a distinct sylvatic ancestor.^33^

The 5′ and 3′ regions of the UTR are well conserved in endemic and sylvatic DENV‐4 strains, suggesting that these UTR regions have important functions, but there are significant differences in the coding regions.^34^ Sylvatic and endemic strains of serotypes 1 and 2 exhibit similar levels of genetic divergence.^35^, ^36^ Nucleotide differences of up to 19% between sylvatic and endemic strains in each dengue serotype have been observed.^16^ This is clearly associated with antigenic variance, and in vitro data suggest that exposure to an endemic DENV serotype provides protection against sylvatic strains from this serotype, while cross‐protection against sylvatic strains from other serotypes is limited.^37^

In general, the rate of DENV evolution may be different between sylvatic and endemic cycles, but there is no conclusive evidence for differences in replication capacity within vertebrates or adaptive evolution between endemic and sylvatic DENV.^12^, ^16^ Therefore, it was suggested that there may be a high risk of sylvatic DENV spreading into human populations and subsequent formation of new endemic strains in humans, but this risk clearly needs further investigation.^12^, ^38^

## 5 THE EVOLUTIONARY DRIVERS OF DENV

### 5.1 Replication errors

Due to the relatively low fidelity of RNA‐dependent RNA polymerases, the number of DENV replication errors is estimated to be around one nucleotide mutation per whole genome replication.^18^, ^39^ The errors caused by these enzymes lead to the accumulation of different genomes in vertebrate hosts, resulting in a population with genetic variation.^18^ However, infection bottlenecks between vertebrate hosts and vectors, which means reductions in viral population size when arbovirus is transmitted between hosts and vectors, have resulted in only a small proportion of the intra‐host variants shaping the inter‐host diversity.^40^, ^41^ As with other flaviviruses, populations of DENV variants within the vector have also been found, which may be due in part to immune mechanisms such as RNAi in the vector.^18^, ^42^ In addition, bottlenecks of vector to host transmission are one of the reasons limiting the spread of variant populations in vectors.^18^ Changes in mosquito population dynamics could further limit the effect of intravector variance in DENV epidemiology, as has been observed for other flaviviruses.^43^

### 5.2 Purifying selection pressure

As an RNA virus, DENV is often subject to strong purifying selection pressure.^11^, ^44^ Interestingly, the extent of the DENV dataset, which contains continent‐wide pandemic data, is comparable to local outbreaks in individual countries.^45^ This is consistent with the fact that the virus is subject to massive negative selection as it switches between vectors and vertebrate hosts.^16^ While it has been suggested that strong purifying selection contributes to lineage extinction and replacement, this was not explicitly demonstrated in a study examining the role of adverse selection in lineage shifts or extinction of variants within a lineage.^46^ Purifying selection has also been shown to affect population variation in humans, but less so on population variation in vectors.^18^

### 5.3 Positive selection pressure

Positive selection was thought to be responsible for the emergence of an epidemic DENV lineage in Puerto Rico.^47^ It was also associated with an A811V mutation in the DENV NS5 protein in clade II of Asian‐American DENV‐2 genotypes from an epidemic in Peru, but it was not accompanied by increased in vitro replication.^48^

Analysis of positive selection pressure on the ancestral DENV strains did not reveal a general and consistent relationship between adaptive evolution and the emergence of the four distinct serotypes.^49^ However, during the divergence of DENV‐2, −3, and − 4 lineages, positive selection occasionally occurred,^50^, ^51^, ^52^ and previous work has shown that there was weak positive selection pressure in these lineages, suggesting that adaptive evolution does indeed affect DENV strain diversity. When interpreting these results, it is important to note that estimates of selection pressure vary considerably between methods and often depend on the constituents of the sequence dataset in detecting statistically significant selection pressure.^53^, ^54^

### 5.4 Lineage shift and replacement

DENV has been repeatedly introduced at different spatial scales, leading to reports of persistence of a single DENV strain in an area.^55^, ^56^, ^57^ These introductions often replace existing clades within genotypes^53^ or serotypes,^58^, ^59^, ^60^ but co‐dispersal of genotypes and serotypes can also occur.^61^, ^62^ New serotypes can also cause bottlenecks and replace previously dominant serotypes,^63^, ^64^ contributing significantly to DENV diversity in a given spatial region.^39^ This pattern of strain introduction, exchange, and migration often limits the long‐term persistence of a given strain in a given region.^65^

In Nicaragua,^66^ Malaysia,^67^ Peru,^48^ Paraguay,^58^ and Sri Lanka,^68^ serotype or subserotype switching coincided with DENV outbreaks, probably because the DENV primed population was infected by a new lineage and more cases with severe symptoms occurred. The expanding epidemic and successful evolution of DENV is thought to result from the increased disease transmission caused by this serotype interaction.^12^ In addition to the effect of prior immunity in populations, other factors have been proposed for DENV lineage shift and displacement, including differences in vector competence,^69^, ^70^, ^71^ viral fitness, host viral loads,^72^ seasonal bottlenecks in vector populations,^12^ human migration,^39^ and random events,^12^ but these hypotheses still need further research. Another hypothesis that requires careful investigation is whether dengue vaccination leads to changes in regional or local DENV population diversity and the adaptive evolution of lineages that escape herd immunity to the vaccine.^73^

### 5.5 Recombination

The possibility that DENV can recombine in the host is plausible and is supported by phylogenetic studies.^74^ For example, during the outbreak on the island of New Caledonia, it was found that one of the patients harbored a mixed infection of DENV‐1, containing viruses assigned to both genotypes I and II, as well as a number of inter‐genotypic recombinants.^74^ Intra‐serotypic recombination of DENV‐1, DENV‐2, and DENV‐3 has been demonstrated in studies, but is relatively rare.^74^, ^75^, ^76^, ^77^ Although coinfection with DENV serotypes has been reported, no recombination between serotypes has been reported.^78^

It is important to note that the presence of recombination in DENV is confirmed only by phylogenetic mismatches in the wild‐type viral sequence and cannot be reproduced in vitro. As a result, the actual frequency of occurrence is controversial.^9^, ^16^ The lack of significant spread and persistence of circulating forms of recombinant DENV suggests that recombinants do not have a significant advantage in viral replication and transmission, and that the effect of recombination is much weaker in the emergence of DENV diversity than in cases of other viruses.^79^, ^80^

## 6 APPLICATION OF THE STUDY OF DENV EVOLUTION TO EPIDEMIC SURVEILLANCE

Phylogenomic analysis of DENV genome can be utilized to improve epidemic surveillance and speed up responses to DENV outbreaks. Phylogeographic analysis is a method that examines the correspondence between phylogenetic and geographic relationships among organisms, thereby clarifying the processes underlying the genetic diversity of populations in space and time. A phylogeographic analysis of the American‐Asian genotype of DENV‐2 in South America suggests that this serotype has spread southward from the Caribbean.^81^ This north–south spreading pattern was also seen in reports of the prevalence of DENV‐1 genotype V and DENV‐3 genotype III in Latin America and can serve as a basis for predicting future routes of DENV strains in this region.^82^, ^83^, ^84^ Globally, DENV genotypes are distributed distinctly across continents. For example, only the American and Asian‐American DENV‐2 genotypes have been found in North and South America, while the Asian‐I DENV‐2 genotype is restricted to Southeast Asia.^16^, ^85^ Thus, some regions may be naive to genotypes that these regions have not been exposed to and surveying these genotypes using sequence data can provide early warning of future epidemiological threats.

Phylogeographic studies can also provide insight into the extent, pathways, and dynamics of transmission within and between different DENV endemic regions. Spatial distribution of DENV has been observed in the Caribbean, and a Bayesian analysis of information from 11 countries in the Caribbean shows that the proximity among these countries is a crucial indicator of cross‐national transmission of DENV strains.^56^, ^86^

Phylogenetic approaches have also provided important information on the spatial distribution patterns of dengue fever at national and smaller scales. Local studies in Thailand have reported the epidemiological dynamics of school cohorts within villages, describing local transmission within and between schools and niche aggregation within households, which can be achieved by analyzing genomic data.^87^, ^88^ A series of DENV infections in Thailand with a micro‐spatial pattern of high resolution has been reported, and it matches with the restricted flight distance of vectors and human movements in many regions with a dengue epidemic, with most DENV infections occurring near patients' homes.^89^ The study suggests that this micro‐spatial pattern was time‐related and that local persistence of DENV strains was not more than half a year, with mixed strains occurring in all parts of Thailand after one dengue season. On the other hand, approximately eight seasons are needed for DENV strains to be mixed thoroughly in other nearby Southeast Asian regions, suggesting that international transmission of the virus is limited. These results contribute to public health interventions as they are useful for predicting the future pathways and dynamics of DENV strains into the tropics.

Using genomic data to parameterize DENV epidemic dynamics over time is important because it provides an estimate of when a new strain of DENV emerged in a particular region and how long it has been circulating before being detected by the surveilling systems. These modern molecular methods can be used to test the reliability of DENV surveillance systems and reveal whether an outbreak is the result of a single infection or co‐infection in a particular location.^90^, ^91^

## 7 LIMITATIONS AND FUTURE DIRECTIONS

Current investigation of the evolution and epidemiology of DENV is largely constrained by the severe lack of sequence data from Africa (according to NCBI GenBank). Given that human cases have been reported since the late 19th century in Africa and that the name of the virus is thought to originate from the Swahili language, Africa may have played an important role in the early emergence of DENV, and addressing the deficiency of sequence information should be a priority issue.^92^ Efforts to reconstruct the early epidemic period of human DENV are limited by the lack of such data.^12^ Recent global efforts to map DENV have shown that DENV circulates in Angola, Gabon, Burkina Faso, Mali, Kenya, and Nigeria,^15^ with severe cases of DENV infection in humans reported in Senegal.^16^, ^93^ To date, all four serotypes have been found in Africa, and there is evidence that these serotypes are becoming more widespread. All four DENV serotypes have also been isolated from sentinel monkeys (and still circulate in the community) in Malaysia, and DENV serotypes isolated from Malaysia and Africa are genetically distinct from each other. Phylogenetic studies have described the presence of a wider diversity of sylvatic DENV strains in Malaysia compared to those in Africa.^16^, ^61^, ^93^, ^94^ In addition, Africa has several sylvatic niches of DENV and a history of viral spill‐over that has resulted in human epidemics of various RNA viruses such as HIV and Ebola.^16^, ^95^, ^96^, ^97^ Thus, there is a possibility that new DENV serotypes may emerge in the region.

Genetic studies of DENV have greatly improved our understanding of the patterns of disease transmission. Further advances in the technology of nucleotide sequencing have made it possible to study DENV strains in natural hosts from the perspective of population, providing opportunities to investigate novel aspects of DENV evolution and epidemiology. New deep sequencing methods can provide a more detailed picture of DENV population size and dynamics at all steps of infection and transmission and contribute to the identification and isolation of sequence variants with a relatively small population.

Little attention has been paid to the ecology and epidemiology of sylvatic DENV, which is critical to understanding and predicting DENV occurrence. Human infection with sylvatic DENV strains has already been observed in West Africa in small epidemics.^37^ Unfortunately, the ecological contact between the sylvatic cycle and the epidemic cycle of DENV has rarely been studied. Further comprehensive and prospective studies of DENV epidemiology and ecology are therefore needed to understand the incidence of DENV epidemics and predict the pattern of DENV re‐emergence.

## AUTHOR CONTRIBUTIONS

All authors meet the requirements for authorship. Xi Yu and Gong Cheng conceived and wrote the manuscript. All the authors read and approved the final version of the manuscript.

## CONFLICT OF INTEREST

The authors declare no competing financial interests. Gong Cheng is the editorial board members of Animal Models and Experimental Medicine and a coauthors of this article. To minimize bias, he was excluded from all editorial decision making related to the acceptance of this article for publication.
